# A Bit Shift Image Encryption Algorithm Based on Double Chaotic Systems

**DOI:** 10.3390/e23091127

**Published:** 2021-08-30

**Authors:** Yue Zhao, Lingfeng Liu

**Affiliations:** School of Software, Nanchang University, Nanchang 330031, China; zhaoyue20210721@163.com

**Keywords:** chaotic system, image encryption, bit shift

## Abstract

A chaotic system refers to a deterministic system with seemingly random irregular motion, and its behavior is uncertain, unrepeatable, and unpredictable. In recent years, researchers have proposed various image encryption schemes based on a single low-dimensional or high-dimensional chaotic system, but many algorithms have problems such as low security. Therefore, designing a good chaotic system and encryption scheme is very important for encryption algorithms. This paper constructs a new double chaotic system based on tent mapping and logistic mapping. In order to verify the practicability and feasibility of the new chaotic system, a displacement image encryption algorithm based on the new chaotic system was subsequently proposed. This paper proposes a displacement image encryption algorithm based on the new chaotic system. The algorithm uses an improved new nonlinear feedback function to generate two random sequences, one of which is used to generate the index sequence, the other is used to generate the encryption matrix, and the index sequence is used to control the generation of the encryption matrix required for encryption. Then, the encryption matrix and the scrambling matrix are XORed to obtain the first encryption image. Finally, a bit-shift encryption method is adopted to prevent the harm caused by key leakage and to improve the security of the algorithm. Numerical experiments show that the key space of the algorithm is not only large, but also the key sensitivity is relatively high, and it has good resistance to various attacks. The analysis shows that this algorithm has certain competitive advantages compared with other encryption algorithms.

## 1. Introduction

Image is a common information carrier with the characteristics of a large data capacity and high redundancy. Despite the rapid development of computer technology and the greatly improved computing speed, the security of digital image transmission has become an increasingly important research topic. However, because of the influence of some attributes of the image itself, the traditional encryption method is no longer suitable for image encryption. In the 1990s, Matthews and Robert proposed a chaotic sequence cipher scheme based on a deformed logistic map [1]. Since then, the concept of chaotic cryptography was proposed and has received widespread attention. Because chaotic systems have good pseudo-random characteristics, unpredictability of orbits, and sensitive dependence on initial conditions, many researchers use it to construct image encryption algorithms [2,3,4,5,6,7,8,9,10,11]. The process of image encryption is usually achieved by changing the pixel positions and pixel values, that is, scrambling and diffusing the pixels of the image.

With the development of image encryption technology, methods for changing pixel positions and pixel values are also emerging. Song et al. introduced a new spatiotemporal chaotic system and proposed an encryption scheme with a scrambling and diffusion mechanism [12]. The diffusion process is carried out pixel-by-bit XOR operation, then a chaos sequence is used to scramble the diffusion result image. The new spatiotemporal chaotic sequence is used for re-diffusion, and finally, an encrypted image is obtained. The analysis of the results shows that the encryption method has a high key sensitivity, large key space, and can resist brute force attacks. Zhang et al. adopted a new bit level displacement algorithm [13] to exchange the pixel low level plane and high-level plane, saving the storage space and improving the efficiency of this algorithm. The diffusion phase is based on the mixed linear-nonlinear coupled map lattice system to complete the encryption process. In [14], Chai et al. proposed a plaintext dependent displacement mechanism within and between the pixels at the same time, which can improve the ability of the algorithm to withstand statistical attacks effectively. Then, the DNA arithmetic rules and a random number diffusion mechanism related to plaintext are used to diffuse the ordinary image. The simulation results show that the method has a good encryption effect.

In general, the chaotic system used for image encryption can be divided into two categories: one is low-dimensional chaotic system, and the other is a high-dimensional chaotic system. Existing low-dimensional chaotic maps, such as logistic mapping, tent mapping, and Arnold mapping, are often favored by researchers because of their simple structure, easy realization, and high efficiency [15,16,17,18,19]. Zhou et al. proposed a symmetric image encryption based on tent mapping [20]. This method uses a new line map based on chaos, which can be applied to pictures of any size and can quickly realize the encryption and decryption of both gray and color images. Yoon et al. introduced a new image encryption algorithm [21] that uses logistic chaotic mapping to generate a small permutation matrix combination in order to generate a large permutation matrix. A large permutation matrix is used to replace the plaintext image, so the randomness of the chaotic map can be effectively propagated to the encrypted image. The experimental results prove that the proposed algorithm can resist statistical cryptographic attacks very well. However, after a series of studies, it was found that low dimensional chaotic systems have problems such as a small key space, poor security, and difficulty resisting brute force attacks [22,23]. In order to overcome the defect of low dimensional chaotic maps, researchers began to explore high dimensional chaotic systems. Compared with the low dimensional mapping system, the high dimensional chaotic mapping has a wide range of parameters, more complex chaotic behavior, and good randomness. Therefore, an image encryption scheme based on the high dimensional chaotic system has been proposed by many researchers [24,25]. Wang et al. proposed a six-dimensional hyperchaotic image encryption algorithm based on bit level alignment and a DNA plane [26]. The key stream generated by the hyperchaotic system is associated with clear images, and then horizontal displacement and DNA coding are used to ensure better security of the cryptosystem, which makes the encryption algorithm more secure. In [27], ElKamchouchi et al. proposed a new bijective image encryption algorithm. This algorithm is a symmetric cryptographic system based on the diffusion phase and the DNA confusion phase of the hyperchaotic system. Using a hyperchaotic system, the algorithm has a large key space, good sensitivity, and high anti attack ability. Although the high-dimensional chaotic system has the advantages of a high complexity and large key space, the complex high-dimensional chaotic system has to face the problems of low efficiency and high consumption.

In recent years, in order to make up for the shortcomings of low-dimensional and high-dimensional chaotic systems, some researchers have used various methods to construct some double chaotic map systems. For example, Abdelfatah proposed a new fast double chaotic image encryption scheme [28]. In this scheme, the author combined tent chaos and Chebyshev chaos to construct a double chaotic pseudo-random generator (DCPG). This method can effectively increase the control parameters of the system and increase the key space of the algorithm, so as to improve the anti-violence attack ability of the scheme. In order to solve the problem of low security in traditional chaotic image encryption, Li et al. proposed a dual chaotic image encryption algorithm based on fractional Fourier change [29]. Experimental results show that the improved algorithm has large key space, reduces the computational complexity, and has good security. Safi et al. proposed an image encryption algorithm based on double logistic mapping [30]. The analysis of the experimental results shows that the use of double logistic chaotic mapping can alleviate the problems of blank windows, small key space, and uneven sequence distribution in a single logistic, so as to obtain a larger chaotic behavior space. Compared with a logistic encryption algorithm, this algorithm has better security. Fu et al. proposed an image encryption transmission system based on a DNA algorithm and double chaos system [31]. The introduction of a double chaotic system can avoid the disadvantage of a small period of low dimensional chaotic mapping. The test results show that the algorithm has a good resistance to attack. Therefore, the chaotic system constructed by the combination of two chaotic maps can make up for the shortcomings of a single low-dimensional or high-dimensional chaotic system, and shows a better chaotic performance.

At present, many image encryption algorithms rely on a single low-dimensional or high-dimensional chaotic system, but there is still little research on image encryption algorithms based on a double chaotic system. The main contribution of this paper is to construct a double chaotic mapping system using the combination of tent mapping and logistic mapping. The new chaotic system is analyzed with the Lyapunov exponent, bifurcation diagram, approximate entropy, and permutation entropy. The results show that compared with tent mapping and logistic mapping, the improved chaotic map exhibits better chaotic characteristics. Based on the new chaotic system, we propose a shift bit image encryption algorithm. The algorithm uses an improved new nonlinear feedback function to generate two pseudo-random sequences, one is used for index sequence generation, and the other sequence selects and converts the value corresponding to the index position according to the generated index sequence, and finally forms an encryption matrix. This sequence conversion method can effectively reduce the correlation between the sequences and can improve the safety of the sequences. The scrambling stage adopts a scrambling method based on row-column transformation. Through this scrambling method, the scrambling of plaintext pixels can be correlated with the number of iterations to fully disrupt the pixel positions and improve the strength of the algorithm. Then, XOR scrambles the image with the encryption matrix to obtain the first encrypted image. In order to prevent the ciphertext after the key is obtained from being deciphered and to improve the security of the algorithm, this paper also proposes a method of bit shift conversion. Finally, the required encrypted image is obtained through bit shift transformation. Statistics and security tests show that the algorithm has sufficient security and has certain anti-attack capabilities.

This article details the generation, encryption, and testing methods of the system. The Section 2 introduces the basic method and improved method of chaotic sequence generation. In Section 3, the methods of the image shuffling algorithm and bit shifting table are introduced, as well as a detailed description of the encryption and decryption steps. The encryption steps are described in detail in a flowchart. In Section 4, some security tests, including statistics, correlations, information entropy, differential attacks, robustness tests, and computation complexity tests are provided for gray histograms. Finally, Section 5 concludes this paper.

## 2. Generation of the Chaotic System

This section mainly describes the generation of chaotic system and the process of generating a pseudo-random sequence based on the new chaotic system. Firstly, the typical tent mapping and logistic mapping are reviewed, and then a new double chaotic system is constructed based on tent mapping and logistic mapping. A series of chaotic performance experiments show that the system has a better chaotic behavior and higher complexity. Finally, the random sequence generation process of the chaotic system is introduced in detail. The experimental results show that the improved chaotic sequence has a good randomness.

### 2.1. Tent Map

The tent map is a piecewise linear 1D chaotic map [32]. It has the characteristics of the simplicity of form, power spectrum density uniformity, and good relevance, etc. It uses the following equation:

(1) $$x_{i+1}=\left\{\begin{array}{cc} \frac{x_{i}}{p} & if\;x_{i}\epsilon \left(0,p\right]\\ \frac{1-x_{i}}{1-p} & if\;x_{i}\epsilon \left(p,1\right] \end{array}\right.$$

**Figure 1:**
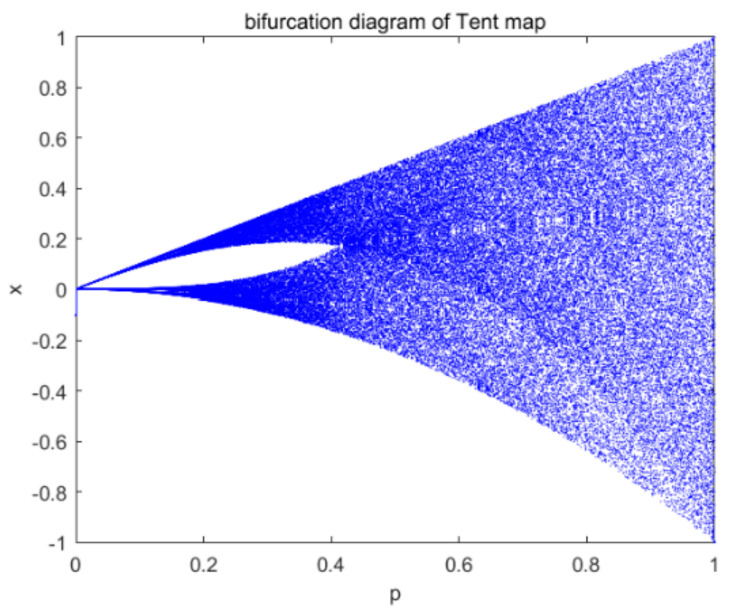
Bifurcation diagram of the tent map.

When *p* is close to 0.5, the form of the tent map becomes most typical. For different parameters, there is a uniform distribution and an approximately uniform distribution density for the sequence. Figure 1 shows bifurcation diagram of the tent map.

Researchers have pointed out that tent mapping can be considered a linear stretching, which is a repeated linear folding process. Linear stretching causes the index of the adjacent points to split, resulting in an initial value sensitivity; it also guarantees the boundedness of the sequence. Some inherent fixed points of iteration have lost their corresponding meaning because of the limited computational precision of a computer.

Although tent mapping is easy to implement, the disadvantages are evident. The results of the piecewise linearity produce a strong correlation of adjacent points. Therefore, many researchers have improved the tent sequence to enhance its security.

### 2.2. Logistic Map

**Figure 2:**
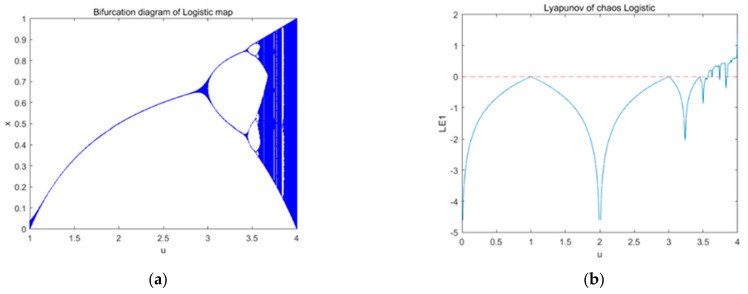
Logistic map: (**a**) bifurcation diagram and (**b**) Lyapunov exponent.

In 1976, American biologist Robert M. Kay put forward logistic chaotic mapping, also known as the bug model [33]. The mathematical form of the logistic mapping is pretty simple, but its dynamic behavior is extremely complex, so it has been very popular in the field of confidential communication and has been widely used. Its formula is as follows:

(2) $$x_{i+1}=\mu x_{i}\;\left(1-\;x_{i}\right)$$

here, *μ* represents the control parameter of logistic map. In Figure 2, we see the bifurcation diagram and the Lyapunov exponent distribution of the logistic map. The diagram shows that when *μ ϵ* (3.56, 4), the sequence is in a state of chaos.

Logistic mapping and tent mapping are topologically conjugated. In a sense, the values of the two maps are identical. Therefore, the chaotic sequences generated by logistic mapping also have strong correlations with the adjacent points. Many researchers have proposed different approaches to improve logistic mapping.

### 2.3. New Chaotic Sequence Generation Algorithm Based on Logistic and Tent Mapping

#### 2.3.1. Sequence Generation Algorithm

In the previous section, the shortcomings of tent and logistic mapping were briefly introduced. For example, the sequences they generate generally have a strong correlation. In addition, they also have problems such as a small key space and poor security. To compensate for these shortcomings, in order to achieve a better ergodicity and pseudo-randomness, a new chaotic sequence generation algorithm is proposed. Its expression is as follows:

(3) $$\left\{\begin{array}{l} x_{i+1}=\left\{\begin{array}{cc} \left(\left(\frac{y_{i}}{p}\;mod\;1\right)+u*\sin\left(\pi x_{i}\right)\right)\;mod\;1 & if\;x\epsilon \left(0,p\right]\\ \left(\left(\frac{1-y_{i}}{1-p}\;mod\;1\right)+u*\cos\left(\pi x_{i}\right)\right)\;mod\;1 & if\;x\epsilon \left(p,1\right] \end{array}\right.\\ y_{i+1}=[\mu x_{i}\left(1-x_{i}\right)+u*\tan\left(\pi y_{i}\right)]mod1 \end{array}\right.$$

**Figure 3:**
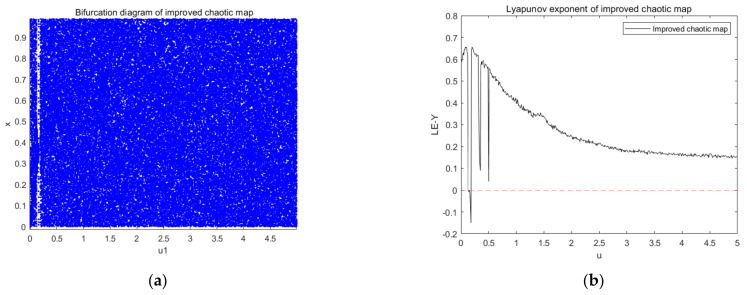
Improved chaotic mapping: (**a**) bifurcation diagram and (**b**) Lyapunov exponent diagram.

Figure 3 shows the bifurcation diagram and Lyapunov exponent of a new type of chaotic map improved by logistic mapping and tent mapping. The distribution of variables in Equation (3) in Figure 3a is more chaotic and disordered in comparison with the previous logistic and tent maps, indicating that the improved chaotic map has better random-like characteristics. Figure 3b shows the distribution of the Lyapunov exponents, and Equation (3) will be chaotic when parameter u locates in a suitable interval. To make Equation (3) have a better performance, we always set u in the interval (0.3, 5) because of its relatively large Lyapunov exponent. It can be seen that the parameter range of the improved chaotic system was much larger than that of the original chaotic map.

In order to prove the complexity of the new chaotic system, the approximate entropy and permutation entropy of the chaotic system were also experimentally analyzed. Approximate entropy (ApEn) is a statistical method used to measure the complexity of a sequence. It uses a non-negative number to represent the complexity of a time series, reflecting the probability of generating new information in the time series. If the entropy of the time series is larger, the randomness of the corresponding time series is stronger, and the probability that new information can be generated is greater. Permutation entropy (PE) is also an indicator to measure the complexity of a time series. It detects dynamic changes in a time series by comparing the values of the adjacent time series. The smaller the permutation entropy, the more regular the chaotic sequence, and the smaller the corresponding complexity. If the permutation entropy is larger, it indicates that the chaotic sequence is more random and the sequence complexity is greater.

**Figure 4:**
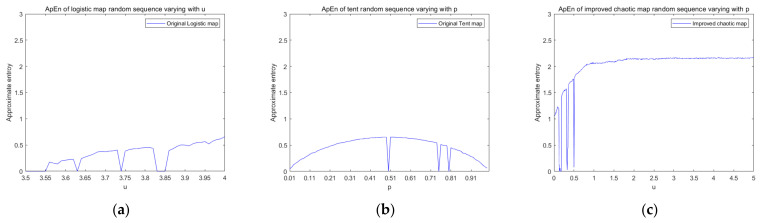
Approximate entropy. (**a**) The ApEn of the logistic random sequence varies with parameter *μ*. (**b**) The ApEn of the tent random sequence varies with parameter *p*. (**c**) The ApEn of the improved random sequence varies with parameter *μ*.

Figure 4 is a graph showing the changes in the approximate entropy of the control logistic chaotic map, tent chaotic map, and improved chaotic map with parameters. From the experimental results, the approximate entropy value of the sequence generated by the improved new chaotic mapping in the chaotic range is greater than the sequence generated by the logistic and tent mapping, and the approximate entropy value tends to be stable, so the new chaotic mapping sequence has a higher complexity.

**Figure 5:**
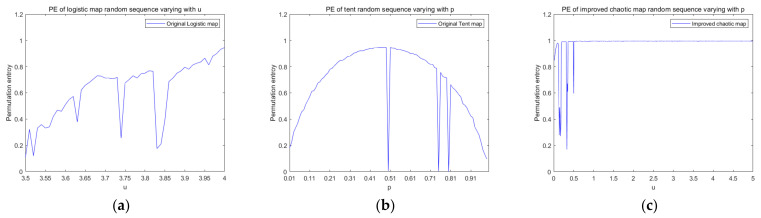
Permutation entropy. (**a**) The PE of the Logistic map random sequence varies with the parameter *μ*. (**b**) The PE of the tent random sequence varies with the parameter *p*; (**c**) The PE of the improved random sequence varies with the parameter *μ*.

Figure 5 is a graph comparing the permutation entropy of these three chaotic maps as a function of the parameters. From Figure 5a, we can clearly observe that with the increase of the parameters of the logistic map, although the permutation entropy shows an upward trend, its variation range is relatively large and the value is less than 1. Figure 5b shows us that the permutation entropy of the tent map tends to decrease as the parameter increases, and the value of the permutation entropy is always small. The permutation entropy of the improved chaotic map in Figure 5c always presents a horizontal straight-line distribution with parameter u in the interval of 0.3 to 5, and the permutation entropy value approaches 1, indicating that the chaotic sequence generated by the improved chaotic map has a high complexity and produces a chaotic sequence with a better randomness. Therefore, compared with logistic mapping and tent mapping, the complexity of the improved chaotic mapping is higher, and the complexity tends to be stable.

The sequence based on improved chaotic map sequences is generated as follow steps:

1. Using formula Equation (3), two pseudo random sequence *x_i_* and *y_i_* are generated with given parameters and initial values.
2. Pseudo random sequence *z_i_* is generated by control sequence *y_i_*, so as to control the *z_i_* sequence generation. The following is the formula for generating the index sequence and the formula for generating the sequence *z_i_* using the index sequence:
  (4) $$idx=floor\left(\left(y\left(i\right)*k\right)\;mod\;65536\right)+1$$
  (5) $$z_{i}=x_{idx}$$
3. Finally, *z_i_* is used to generate the integer sequence *z_m_*, needed for encryption. The specific formula is as follows:
  (6) $$z_{m}=z_{i}\;\mathrm{mod}\;256$$

By using the above formula, the sequence can be disrupted effectively, the strong correlation between sequences can be eliminated, and it can improve the security of sequences; therefore, the security of encryption is guaranteed.

#### 2.3.2. Randomness Test of the Generated Sequence

**Figure 6:**
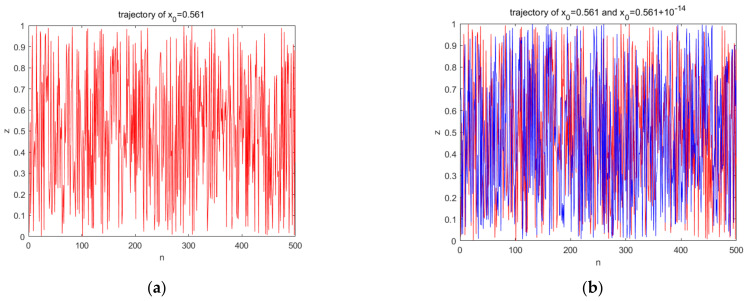
Trajectories of the generated sequence: (**a**) trajectory of *x*_0_ = 0.561, and (**b**) trajectory of *x*_0_ = 0.561 and *x*_0_ = 0.561 + 10^−14^.

**Figure 7:**
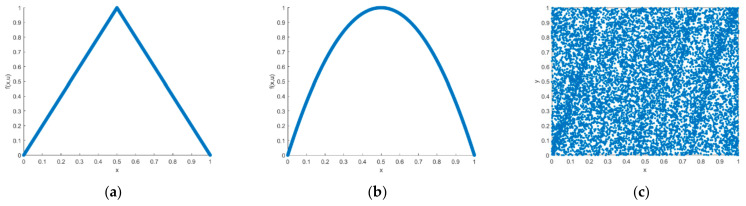
Attractors of different chaotic systems: (**a**) tent mapping, (**b**) logistic mapping, and (**c**) improved chaotic mapping.

A good chaotic sequence does not have a certain period and has characteristics sensitive to the initial values. When the initial value was set to $x_{0}=0.561,y_{0}=0.458,\;\mu =4.0,u=2,\;p=0.501,k=100,000,$ the trajectory was as shown in Figure 6a. When the initial value was slightly changed, the two iteration trajectories were as shown in Figure 6b. Therefore, from the perspective of trajectory, the improved chaotic sequence had a good pseudo-randomness. The visible sequence had an initial value sensitivity and no periodicity. Figure 7 is a chaotic attractors graph of the different chaotic systems; from the figure, we can clearly see that the attractors of tent mapping and logistic mapping show a certain regular distribution, while the distribution of the attractors of the improved chaotic system is messy, which also shows that the improved chaotic sequence has a better randomness.

#### 2.3.3. NIST Test

Furthermore, to detect the randomness of the generated sequence, we used the National Institute of Standards and Technology (NIST) statistical test. NIST randomness testing uses the method of probabilistic statistics for describing the pseudo-randomness of sequences that are generated with a random number generator or cryptographic algorithm. Describing the disparity of the sequence being tested with the real random sequence from various perspectives for different test items is generally done using the method of hypothesis testing. In a random hypothesis test, an aspect of a known true random sequence conforms to a particular distribution. It is assumed that the sequence to be detected is random, and in this should also conform to this special distribution.

In this study, 200 groups of different sequences with different parameters were tested. Then, the number of passes in these tests were recorded to calculate the pass rate for each test.

Table 1 reveals a pass rate of more than 98%, from which it can be indicated that the sequence had good pseudorandom characteristics.

### 2.4. Selection of Parameter k

In this research, the parameters to be set were the logistic-mapped control parameter μ, the tent-mapped control parameter p, the trigonometric range control parameter u, and the position selection control parameter k. Among these parameters, μ, p, and u as well as the initial values $x_{0}\;\mathrm{and}\;y_{0}$ were always used as the secret keys, while *k* was fixed.

**Figure 8:**
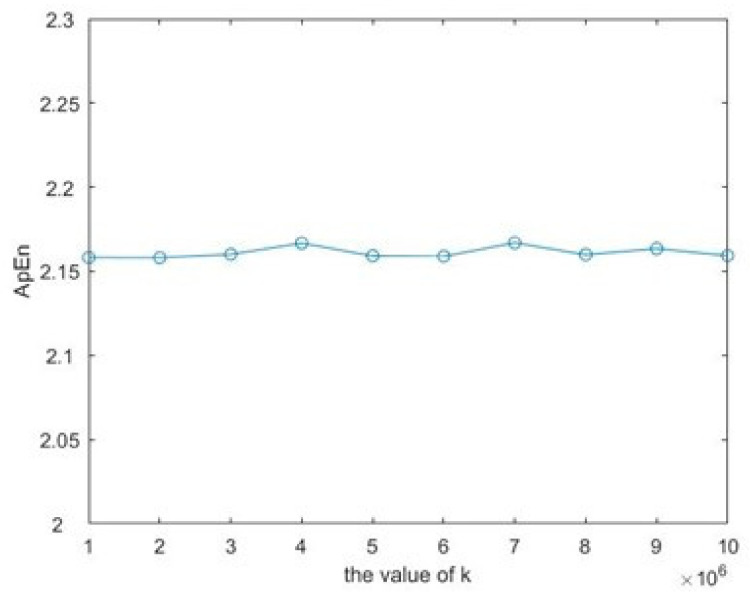
ApEn with different k.

For parameter k, the chaos degree of the generated sequence could be measured by the approximate entropy in order to determine the optimal *k* value. Approximate entropy (ApEn) is the probability of discovering new subsets’ generation over time, and it is the measure of the complexity of the unstable time series. ApEn can be used to describe the irregularity of complex systems. For sequences generated by chaotic systems, the higher the random value, the larger the approximate entropy. Conversely, the lower the random value, the smaller the approximate entropy. As Figure 8 shows, ApEn does not fluctuate much when k is taken as 10^6^. Therefore, in this paper, we set *k* = 7 × 10^6^ all through the paper if not specified.

To sum up, the double chaotic system proposed in this paper has better a chaotic performance than the original logistic map and tent map, and the improved chaotic sequence has better randomness. In order to verify the practicability of the double chaotic system, a bit shifted image encryption algorithm was also proposed in this paper. The details are shown in the following section.

## 3. New Algorithm Based on the New Chaotic System

**Figure 9:**
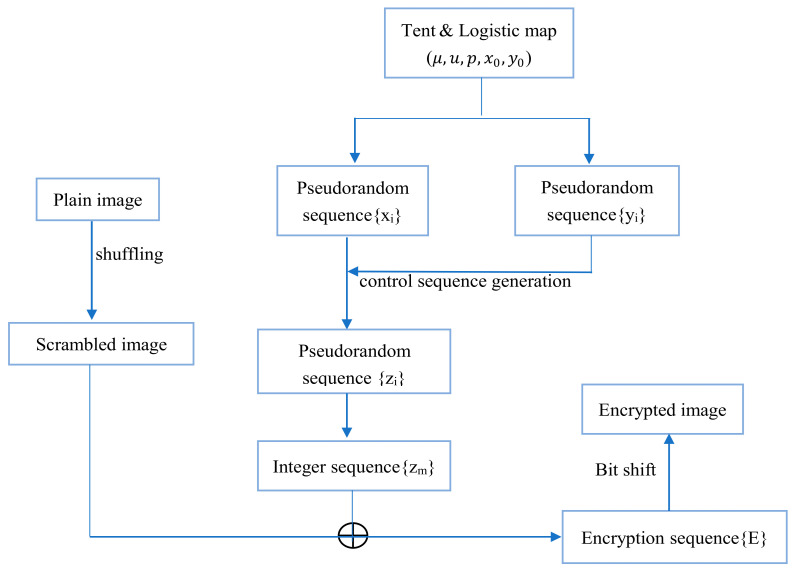
The flowchart of encryption algorithm.

The flowchart of the new image encryption proposed in this paper is shown in Figure 9. It can be seen from the figure that the algorithm can be divided into four stages in detail, namely the scrambling stage, sequence generation stage, XOR operation stage, and bit shift stage.

### 3.1. Image Shuffling

In order to scramble the position of the pixels, this paper adopts a scrambling method based on row-column transformation, which can improve the security and strength of the algorithm against noise and clipping attacks. Let the pixel value of the *i*th row and the pixel value of the *j*th column of the plaintext image be represented by *i* and *j*, respectively. For each iteration, the corresponding row or column must be located and then exchanged. For iteratively n times, the position of the pixel moves to *p* row and *q* column. One of the iterative formulas is as follows:

(7) $$\left\{\begin{array}{l} p=\left(i^{2}\;mod\;256\right)+1\\ q=\left(n*j\;mod\;256\right)+1 \end{array}\right.$$

After generating *p* and *q*, swap the *i*th row and *p*th row, as well as the *j*th column and *q*th column, of the original image and finally get the scrambling image.

In this manner, scrambling the pixels of the plaintext can be associated with a specific number of rounds for the pixel positions to be sufficiently scrambled, thereby effectively improving the strength of the algorithm.

### 3.2. Bit Shift Transformation

If the attacker obtains the key, the encryption algorithm can completely decipher the acquired plaintext image. After the cipher matrix and the scrambled plaintext matrix are XORed, a block shift is performed to prevent the ciphertext from being deciphered after the key is obtained. The transformation method is more flexible and can be changed according to the needs of the users. The shift transformation method adopted in this algorithm is shown in Table 2.

The encrypted ciphertext image can be further divided into blocks and then nonlinearly transformed via bit shifting, thereby improving the security of the encryption. The matrix is first divided into rows and columns of 4 × 4 size blocks. Then, each block is transformed and shifted according to the shift transformation method in Table 2. For example, to shift the first block, that is, the first row and first column in the table, the corresponding shift digit is 4, which is to move all the elements in the first block circularly to the left by 4 bits to get the final shift position. Similarly, the number of shift bits corresponding to the first column of the second row is 5, that is, the element moves 5 bits to the left for shift transformation, and so on, until all the steps of the shift transformation are completed.

### 3.3. Encryption Algorithm

In order to enhance the correlation between the plaintext image and the encryption algorithm, resistance to selected plaintext attacks needed to be improved, so the initial value of the chaotic system used in the encryption algorithm process was determined by the plaintext image. This algorithm used the pixel average value of the plaintext image to update the initial values *x* and *y* of the chaotic system. The key required for encryption is the control parameter of the chaotic map and the initial values of *x* and *y* of the chaotic sequence. Next, the encryption steps of this encryption scheme will be introduced in detail.

Step 1: Read plaintext image *P* (in the test, the image size is uniformly set to 256 × 256). Use the plaintext image *P* to calculate the initial value of the chaotic map, *x* = aver(P)/255, *y* = 1 − *x*.

Step 2: Use the scrambling algorithm steps introduced in Section 3.1 to scramble the read plaintext image *P* to obtain a scrambled image *P*′.

Step 3: According to the chaotic Equation (3) and the initial values of *x* and *y* of the chaotic sequence generated in Step 1, the chaotic sequence {*x_i_*} and the control generation sequence {*y_i_*} are generated iteratively.

Step 4: Generate an index sequence {*idx*} using Equation (4) in the sequence generating of Section 2.3.1.

Step 5: The {*idx*} generated in Step 4 is used to generate the encryption sequence {*z_i_*} according to Equation (5).

Step 6: Bring the chaotic sequence {*z_i_*} in Step 5 into Equation (6) to obtain the encrypted integer sequence {*z_m_*}.

Step 7: Then, the encrypted sequence in Step 6 is XORed with the scrambled image *P*′, and the encrypted image *E* can be obtained.

Step 8: The image *E* obtained in Step 7 is subjected to a block bit shifting operation according to the bit shifting method shown in Table 2 in Section 3.2, so as to obtain the final encrypted image *E*′.

### 3.4. Decryption Algorithm

This section mainly describes the decryption process in detail. After the receiver obtains the encrypted image *E*′ and key *K*, then the decryption is performed according to the following steps.

Step 1: Read the ciphertext image *E*′.

Step 2: Shift image *E*′ according to the shift mode in the shift table to get the decrypted image *E* after the first step.

Step 3: Follow Steps 3–6 in the encryption algorithm to generate the chaos sequence {*x_i_*}, control generating sequence {*y_i_*}, indexes sequence {*idx*}, chaotic sequence {*z_i_*}, and ultimately decryption sequence {*z_m_*}.

Step 4: XOR the image *E* obtained in Step 2 with the decryption sequence {*z_m_*}, and finally obtain the scrambled image *P*′.

Step 5: Perform the scrambling inverse operation on the image *P*′ obtained in Step 4 according to the scrambling Equation (7) in order to obtain the plaintext image *P*, and complete the entire process of image decryption.

## 4. Experimental Results and Safety Analysis

This section mainly tests the security of the ciphertext image, and compares it with the research results of the other researchers. The simulations were run on a 2.3 GHz CPU laptop by using MATLAB R2018a. In the test, if there was no special statement, the keys were always set to *μ* = 4.0, *u* = 2.0, *p* = 0.501, *k* = 100,000, *x* = aver(*M*)/255, y = 1 − *x*, where aver(*M*) presents the average pixel value of the plaintext image *M*, which strengthens the ability to resist differential attacks.

### 4.1. Analysis of Experimental Results

**Figure 10:**
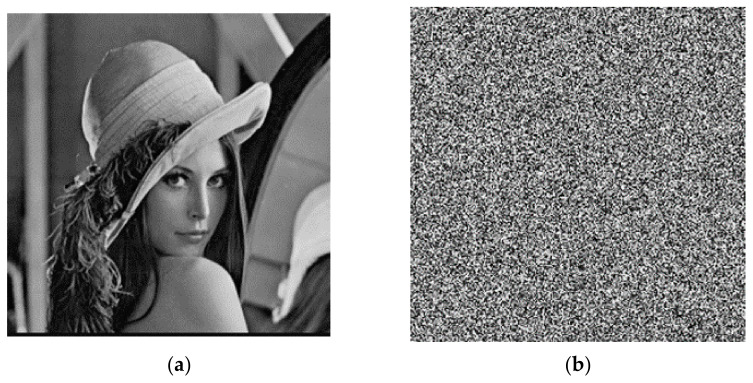

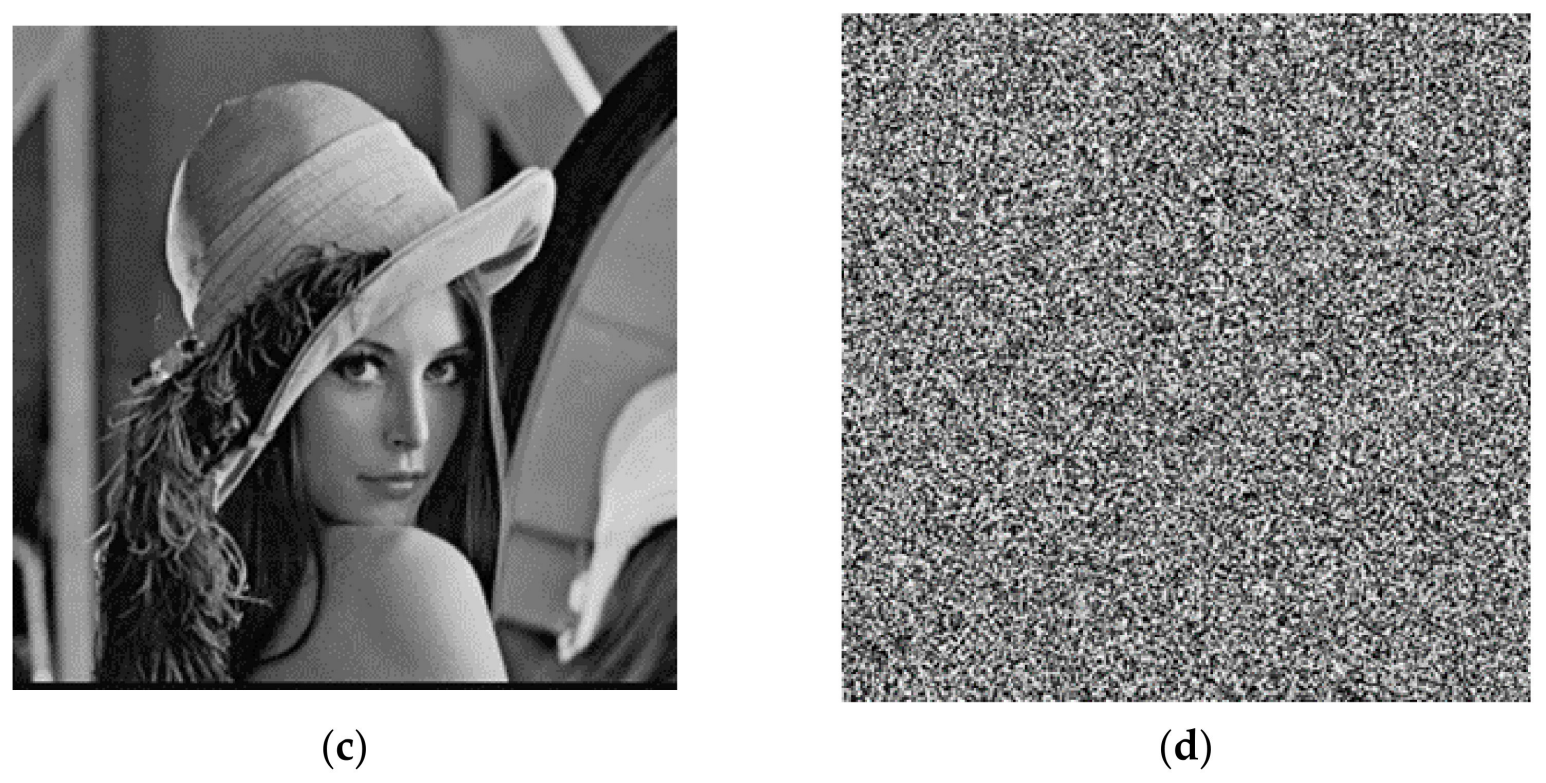
Encryption and decryption test: (**a**) original image; (**b**) encryption image; (**c**) correct decryption image; (**d**) error decryption image.

The plaintext image needs encryption keys to encrypt. Then, it can decrypt the image separately with the correct decryption key and the wrong decryption key. The results are shown in Figure 10. After encrypting with the key, the distribution of the image has no regularity, and no valid information in the original image is observed. For the decryption operation, if the correct decryption key is used, then the encrypted image can be restored to the plaintext image perfectly; if the decryption key is wrong, the image of the error decryption is also evenly distributed, and it is unable to obtain the valid information in the original image. Therefore, the algorithm of the encryption and decryption has good usability.

### 4.2. Key Space Analysis

The key space size has a direct impact on the performance of the cryptographic algorithm, and a good cryptographic algorithm has to have a sufficiently large key space. If the key space is small, it will be easy for an attacker to use brute force to obtain the correct key. For the purpose of making encryption have a good security, its key space cannot be less than 2^128^.

In this study, the initial values of *x*_0_ and *y*_0_ were also the key to the algorithm, in addition to the control parameters of the logistic map *μ*_0_, tent map *p*, and trigonometric function *a*. The key space of each key was set to 10^14^ to compare it with other researchers’ key space. The calculation of the key space is as follows:

$$key\;space=10^{14}\times 10^{14}\times 10^{14}\times 10^{14}\times 10^{14}=10^{70}\approx 2^{232}\gg 2^{128}.$$

The results of the key space showed a value greater than 2^128^, so the description of the security of the algorithm was good. In Table 3, we also compare our key space with that of other researches. From Table 3 it can clearly be seen that although the algorithm’s key space is smaller than some of the other algorithms, it is larger than the referenced key space [12,17]. Therefore, this algorithm key was large enough, could satisfy a good encryption algorithm the request, and could resist the violent attack.

### 4.3. Grayscale Histogram Analysis

The grayscale histogram is for the gradation level distribution function, and is used for the image gradation level distribution statistics. It reflects the frequency of each pixel gray level appearing in the image in relation to the gray level, with the gray level as the horizontal coordinate and frequency as the vertical coordinate.

In the grayscale histogram of the plaintext image, a large amount of information is hidden in it. Therefore, the distribution of the ciphertext is of vital importance. It should hide the redundancy of plaintext, so that any information that has relations between plaintext and ciphertext will not be disclosed.

**Figure 11:**
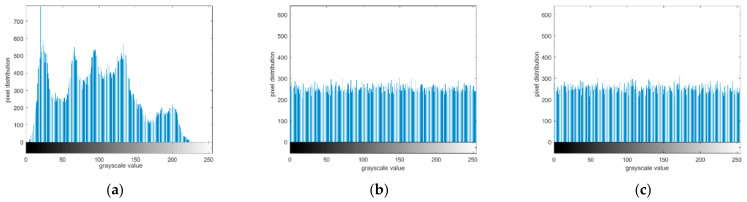
Grayscale histogram: (**a**) Lena; (**b**) encryption with bit shift; (**c**) encryption without bit shift.

During this process, the histogram of plaintext image was first plotted, and then we drew the corresponding histogram of the encrypted image. After encryption, the image histogram was divided into two kinds. One was the ciphertext image histogram before bit shifting, and the other was the encrypted image histogram after bit shifting. According to the gray histogram in Figure 11, regardless of whether displacement was performed, the distribution of the histogram of the ciphertext image was uniform, which is significantly different from that of the plaintext image. Therefore, no attack clue is provided.

### 4.4. Correlation Analysis

In the case of plaintext images, the correlation between each pixel and its adjacent pixels was high in the horizontal, vertical, and diagonal directions. A good algorithm for encryption necessitates ensuring that there is no strong correlation among the adjacent pixels of the encrypted image.

First, 10,000 pairs of pixels were chosen from Lena images for the correlation coefficient calculation. The following is the expression of the correlation coefficient:

(8) $$Cov\left(x,y\right)=\frac{\sum_{i=1}^{n}\left(x_{i}-\bar{x}\right)\left(y_{i}-\bar{y}\right)}{n}$$

(9) $$p=\frac{Cov\left(x,y\right)}{\sigma_{x}\sigma_{y}}=\frac{\sum_{i=1}^{n}\left(x_{i}-\bar{x}\right)\left(y_{i}-\bar{y}\right)}{n\sigma_{x}\sigma_{y}}$$

which is:

(10) $$p=\frac{\sum_{i=1}^{n}\left(x_{i}-\bar{x}\right)\left(y_{i}-\bar{y}\right)}{\sqrt{\sum_{i=1}^{n}{\left(x_{i}-\bar{x}\right)}^{2}}\sqrt{\sum_{i=1}^{n}{\left(y_{i}-\bar{y}\right)}^{2}}}$$

where *x* and *y* are the gray values of two adjacent pixels of the plaintext image or ciphertext image, respectively.

Table 4 shows the value of the correlation coefficient of this algorithm and the other algorithms. As illustrated Table 4, from the perspective of vertical, horizontal and diagonal, the pixel values of the plain text images had a strong correlation, and their values were all greater than 0.9. After encryption, the correlation coefficient between the adjacent pixel values was very low with a value of almost 0. The correlation values of the other researchers’ algorithms were also studied for comparison. By contrast, the correlation coefficient of this adjacent pixel was less than other research algorithms. Therefore, the algorithm in this study has good security.

**Figure 12:**
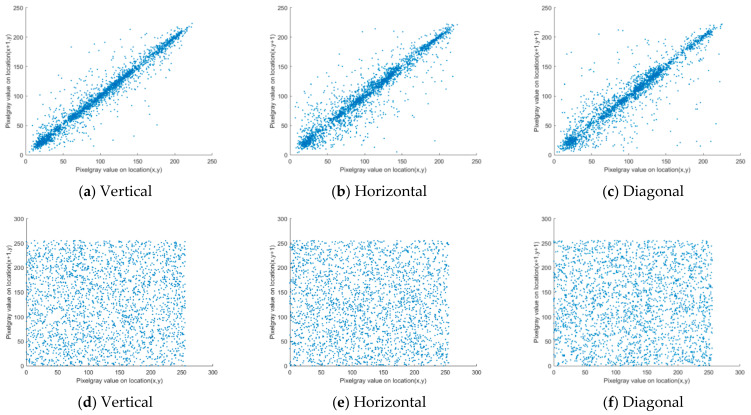
Correlation analysis. (**a**) Correlation of Lena image in vertical direction; (**b**) Correlation of Lena image in horizontal direction; (**c**) Correlation of Lena image in diagonal direction; (**d**) Correlation of encrypted image in the vertical direction; (**e**) Correlation of encrypted images in the horizontal direction; (**f**) Correlation of encrypted images in the diagonal direction.

As shown in Figure 12, for an intuitive observation, the distribution of the pixels adjacent to each other in the plaintext and ciphertext images were plotted separately. The adjacent pixels of the plaintext image were linearly distributed with a significant correlation before the encryption. After encryption, the distribution of the pixel points was scattered, and no law of distribution was observed. Therefore, the algorithm in this study could completely disrupt the original correlation of the image, and has good security and usability.

### 4.5. Information Entropy Analysis

Information quantity is the measure of information, while entropy is the expectation of information quantity before the result comes out. Information entropy can be used as a quantitative standard for evaluating images. Information entropy is considered by the statistical properties of the entire source. It characterizes the overall characteristics of the source. A source has only one information entropy. Different sources have different entropies due to different statistical characteristics. The uncertainty of a variable is greater, the greater its information entropy. The formula for entropy is:

(11) $$H\left(k\right)=-\overset{2^{N}-1}{\underset{j=1}{\sum }}P\left(k_{j}\right)log_{2}P\left(k_{j}\right)$$

For a grayscale image, its gray value should not exceed 255; thus, the smallest value of entropy is 0 and the largest value is 8, which indicates that the higher the entropy of the encrypted image, the higher the security.

The entropy of the information of the plaintext image and the encrypted image is first calculated by formula, and then make a comparison of other researchers’ algorithms. Results illustrated as in the Table 5.

The results show that the entropy of ciphertext is high, which makes the information disclosure difficult. By comparing the experimental data of each algorithm in the above table, can be clearly observed, although the information entropy of the algorithm is less than [19], it is better than that of other researchers.

### 4.6. Key Sensitive Analysis

The key sensitivity also has the very tremendous influence to the algorithm security. This section tests the sensitivity of the encryption and decryption keys separately.

**Figure 13:**
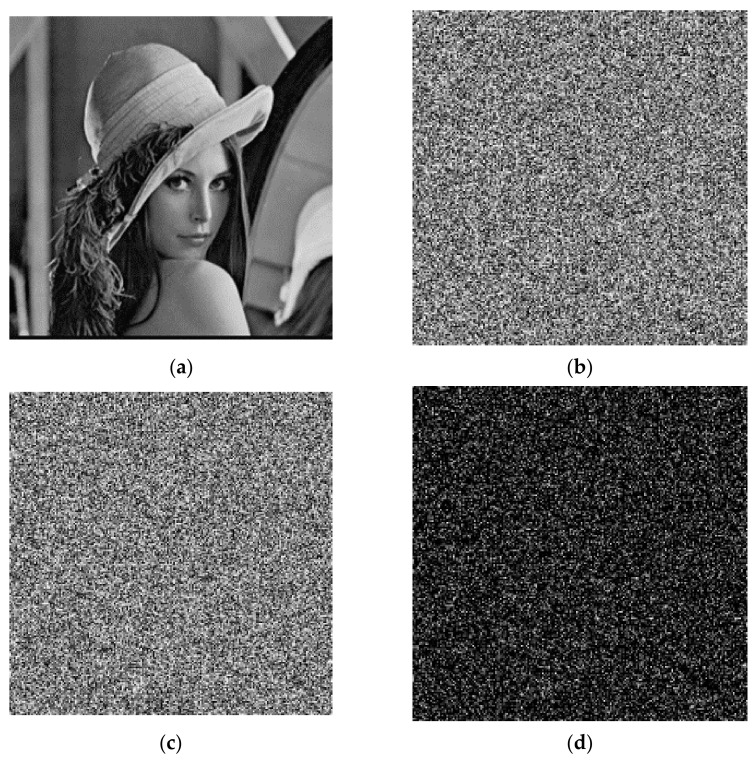
Encryption key sensitive: (**a**) original image; (**b**) *x* = 0.561; (**c**) *x* = 0.561 + 10^−14^; and (**d**) subtraction of two figures.

- Sensitivity of the encryption key;

For the encryption key sensitivity test, in these five keys, only small changes were made to the key *x*. First, the initial value of *x* is set to *x* = 0.561, and *x* = 0.561 + 10^−14^, respectively. Encrypt the “Lena” image with these two sets of keys separately so that we get two encrypted images. The test effect is indicated to Figure 13.

**Figure 14:**
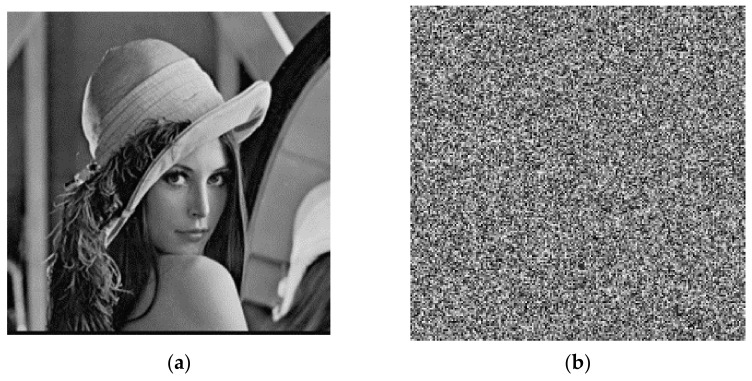

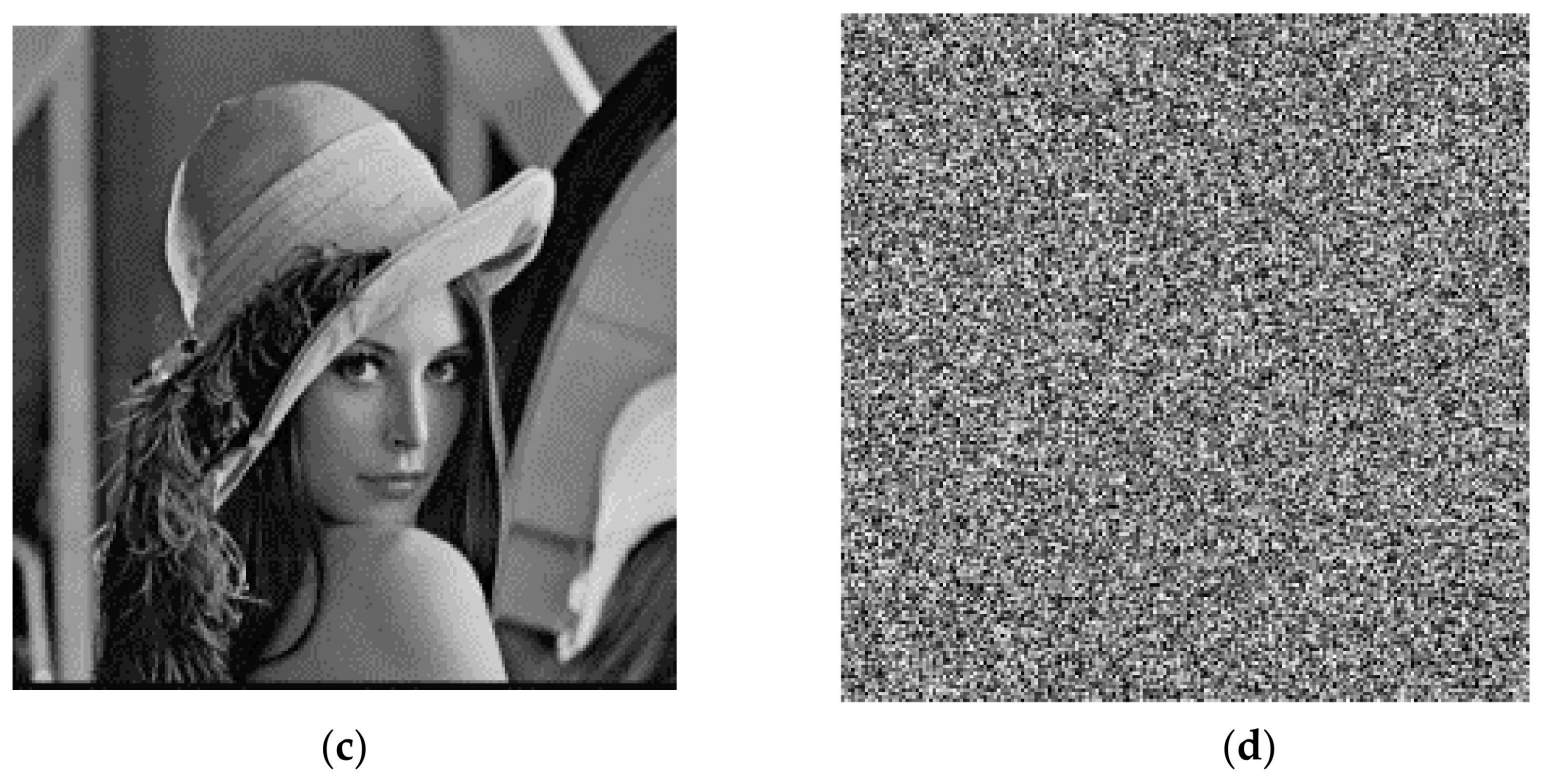
Decryption key sensitive: (**a**) original image; (**b**) encrypted with *x* = 0.561; (**c**) decrypted with *x* = 0.561; (**d**) decrypted with *x* = 0.561 + 10^−14^.

- Sensitivity of the decryption key;

The same sensitivity test was performed on the decryption keys. First, the plaintext image was encrypted with the key *x* = 0.561. The decryption keys *x* = 0.561 and *x* = 0.561 + 10^−14^ were each set, and the decrypted image content was compared twice. The test results are indicated in Figure 14. The test demonstrated that even if the decryption key was changed only very slightly, the decrypted image would change dramatically. Consequently, the decryption key is also highly sensitive.

### 4.7. Differential Attack Analysis

Differential attack is a way to attack the encryption algorithm that is performed via the comparison and analysis of specific differences in plaintext in terms of changes propagated through the encryption.

**Figure 15:**
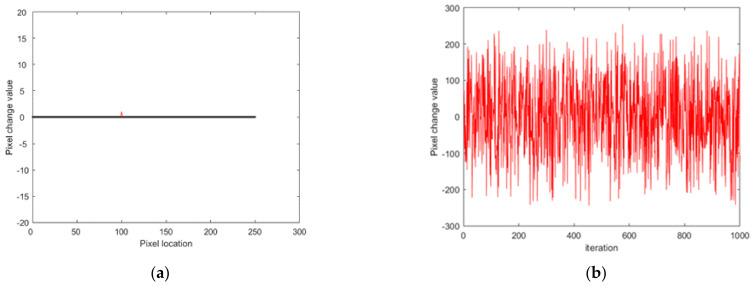
Differential attack: (**a**) before change and (**b**) after change.

The capacity to resist differential attacks is closely related to the sensitive nature of the plaintext image. Figure 15 shows the change in the ciphered image after a slight change in the location of the (100,101) pixel values in the original image.

As shown in Figure 15, even if the pixel value of the original image (100,101) was changed by 1, the encrypted image changed considerably, indicating that the algorithm of this research has a certain sensitivity to the original image.

In addition, the capability of the algorithm to resist differential attacks could also be analyzed by calculating the pixel change rate (NPCR) of the encrypted image and the unified average change intensity (UACI). The most common method to measure plaintext sensitivity is to analyze NPCR and UACI.

The closer the NPCR is to 100%, the more sensitive the plaintext changes corresponding to its encryption system are, making it resistant to differential attacks. The closer the UACI is to 33.33%, the better the differential resistance of the attacks. The following is the formula for calculating the NPCR and UACI values, and the calculation results are shown in Table 6.

(12) $$\mathrm{NPCR}=\frac{\sum_{i,j}D\left(i,j\right)}{W\times H}\times 100\%$$

(13) $$\mathrm{UCAI}=\frac{1}{W\times H}\left[\underset{i,j}{\sum }\frac{\left|c_{1}\left(i,j\right)-c_{2}\left(i,j\right)\right|}{255}\right]\times 100\%$$

As is clear from Table 6, the NPCR and UACI values of the scheme were superior to those of most other researchers’ algorithms. Except for [17], the NPCR values of the other schemes were close to 100%. The UACI value analysis shows that this scheme is superior to [7,13,17], similar to the results of the other references. Therefore, the scheme may well resist the attack difference and meet certain security requirements.

### 4.8. Robustness Analysis

Robustness is the strength of the algorithm, meaning that in abnormal circumstances, the original image can still be restored. This feature has no effect on the use of images. In this test, the intensity of the image was simulated by some changes in pixels as a result of noise during transmission. However, when the image noise pollution is serious or the image is oversized, the decrypted image quality may deteriorate. Thus, it is very significant to analyze the image quality after decryption. The robustness of the algorithm was verified by malicious attacks.

The mean square error (MSE) and peak signal-to-noise ratio (PSNR) are objective standards for evaluating the image quality. MSE reflects the variance between the current image and the source image at each pixel, and its calculation formula is shown in (14):

(14) $$\mathrm{MSE}=\frac{1}{M\times N}\overset{M}{\underset{i=1}{\sum }}\overset{N}{\underset{j=1}{\sum }}{\left(X\left(i,j\right)-Y\left(i,j\right)\right)}^{2}$$

in which *M* × *N* is the original image size, *X*(*i*, *j*) represents the original image, and the ciphertext image is represented by *Y* (*i*, *j*). If the image quality is good, then the MSE value is relatively high.

The PSNR is the ratio of the maximum semaphore to the noise intensity. The PSNR results use Decibel (dB) as the unit. The larger the PSNR value between two images, the more likely it is to have no deterioration. When the degree of deterioration is larger, the PSNR value tends to be 0 dB. Its calculation formula is as follows:

(15) $$\mathrm{PSNR}=10\times \log_{10}\left(\frac{255^{2}}{MSE}\right)$$

**Figure 16:**
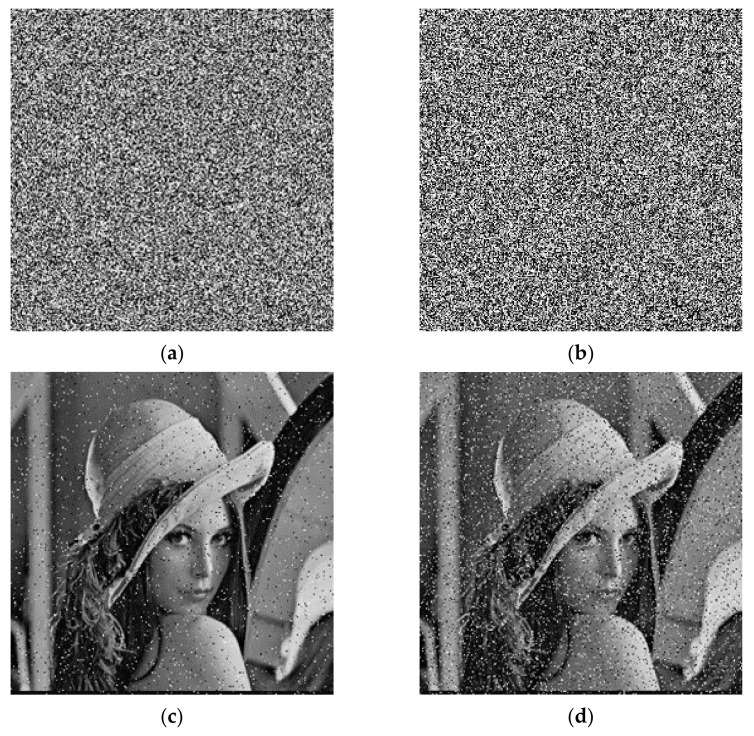
Noise attack: (**a**) 5% salt and pepper noise; (**b**) 15% salt and pepper noise; (**c**) decryption with 5% salt and pepper noise; (**d**) decryption with 15% salt and pepper noise.

For the detection of noise attacks, it is necessary to add noise to the ciphertext image. First 5% and 15% intensity of salt noise and pepper noise, respectively, were added, and then decrypted. The experimental result is illustrated in Figure 16. For additional strength at 5% noise, the PSNR value is 22.3596, and for the noise with the addition intensity of 15%, the PSNR value is 17.5042. It shows that after the image with noise added was decrypted, the image as a whole did not undergo drastic changes and could still be used normally. Thus, the algorithm has a certain capacity to anti-noise attacks.

**Figure 17:**
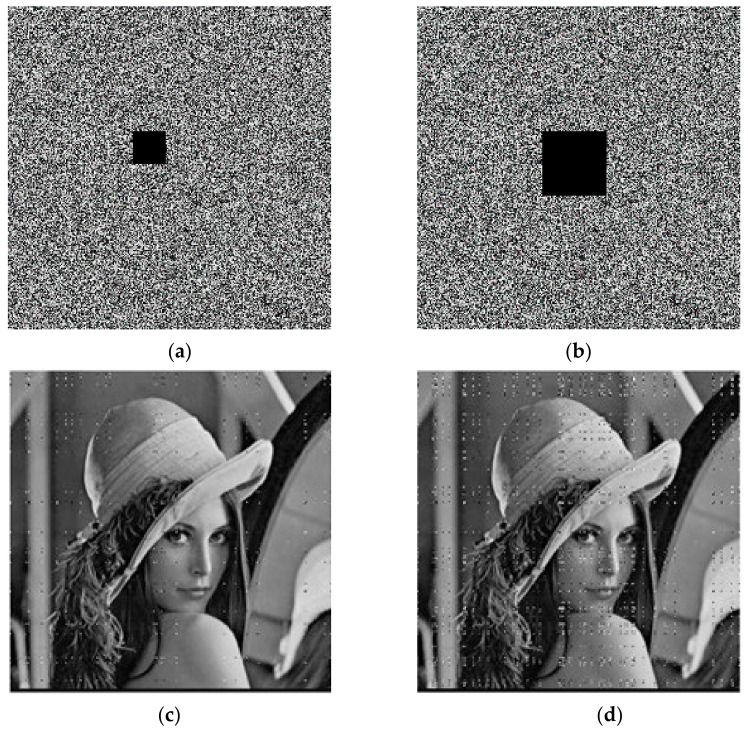
Cropping attack: (**a**) 0.95% cropping; (**b**) 3.81% cropping; (**c**) decryption with 0.95% cropping; (**d**) decryption with 3.81% cropping.

For the clipping attack, the ciphertext images reduced by 0.95% and 3.81% were decrypted, separately. The results are illustrated in Figure 17. For the ciphertext images reduced by 0.95%, the PSNR value was 29.4360, and for the ciphertext images reduced by 0.95%, the value of PSNR was 23.3324. From this, we can see that after the cropped ciphertext image was decrypted, the overall image did not change much; it still had a high recognition and could be used normally. Therefore, this algorithm can resist clipping attack.

### 4.9. Computation Complexity Analysis

Encryption speed directly affects the performance of encryption, so it is also critical for cryptographic algorithms. In this experiment, the encryption algorithm ran on a 2.3 GHz CPU with 8 G main memory. The results (in Table 7) show that the encryption algorithm in this study is competitive compared with the other chaotic encryption algorithms.

## 5. Conclusions

This paper proposes a double chaotic system based on logistic mapping and tent mapping. Two sequences are generated using a new chaotic map: one to generate the index sequence, and the other is to get the final chaotic sequence using the generated index sequence. The pseudo-random of the sequence is verified by detecting the sequence trajectory and attractor. The NIST test also illustrates that the sequence has a good pseudorandom characteristic. The security of the encrypted images is also enhanced by bit shifting. The correlation experiment indicates that the encryption algorithm is effective at breaking the correlation between the adjacent pixels and is well resistant to statistical attacks. In this study, the algorithm can resist various forms of violent attacks thanks to its large enough key space, and it also has a strong robustness. These are sufficient to prove that the encryption algorithm can ensure the security of the encryption, and has a high feasibility. The effect of image encryption is remarkable.

Finally, in subsequent research, some new image encryption algorithms can be combined with compressed sensing, DNA, and other theories, so as to reduce the pressure of image transmission, and to achieve efficient and secure image encryption.

## Figures and Tables

**Table 1:** NIST tested.

Test Name	Pass Rate (%)	Results
Frequency	99.58	success
Block Frequency (m = 128)	98.97	success
Cumulative Sums Forward	99.32	success
Cumulative Sums Reverse	99.69	success
Runs	99.97	success
Longest Run of Ones	99.13	success
Rank	98.59	success
FFT	99.94	success
Non-Overlapping Template (m = 9, B = 000000001)	99.22	success
Overlapping Template	98.89	success
Universal	99.88	success
Approximate Entropy	99.32	success
Random Excursions (x = 4)	98.76	success
Random Excursions Variant (x = 4)	98.84	success
Linear Complexity	99.33	success
Serial	98.86	success

**Table 2:** Shift transformation method.

	Column	1	2	3	4
Row					
1		4	5	1	2
2		3	7	2	6
3		1	3	3	7
4		5	2	7	4

**Table 3:** Key spaces.

Algorithm	Key Space
Proposed algorithm	2^232^
[8]	2^293^
[12]	>2^100^
[17]	2^192^
[18]	2^256^
[34]	2^256^
[35]	2^332^

**Table 4:** Correlation coefficients.

Algorithm	Horizontal	Vertical	Diagonal
plaintext	0.9716	0.9318	0.9095
proposed algorithm	−0.0015	−0.0032	0.0023
[7]	0.0011	0.0078	0.0027
[8]	0.0066	−0.0081	0.0077
[12]	0.0055	0.0041	0.0002
[18]	0.0015	0.0043	0.0023
[19]	0.0035	0.0037	−0.0095
[21]	−0.0070	−0.0150	0.0030
[26]	0.0085	0.0054	0.0049
[27]	0.0082	−0.0032	−0.0025
[35]	0.0026	−0.0004	−0.0002

**Table 5:** Information Entropy.

Algorithm	Information Entropy
Lena	7.5534
Proposed algorithm without bit shift	7.9993
[8]	7.9993
[12]	7.9971
[17]	7.9993
[19]	7.9999
[26]	7.9975
[27]	7.988
[35]	7.9973

**Table 6:** Values of NPCR and UACI.

Algorithm	NPCR, %	UACI, %
Proposed algorithm	99.68	33.58
[7]	99.66	29.28
[8]	99.59	33.40
[12]	99.65	33.52
[13]	99.58	30.78
[17]	75.63	34.94
[26]	99.63	33.47
[27]	99.62	33.42
[35]	99.60	33.50

**Table 7:** Computation complexity test.

Algorithm	Time (s)
Proposed algorithm	0.013
[13]	0.022
[34]	0.080

## Data Availability

The data presented in this study are available on request from the corresponding author.
